# 2,2′-[1,5-Bis(4-amino­phen­yl)-1,5-dihydro­benzo[1,2-*d*;4,5-*d*′]diimidazole-2,6-di­yl]diphenol

**DOI:** 10.1107/S1600536811036737

**Published:** 2011-09-17

**Authors:** Anita Blagus, Branko Kaitner

**Affiliations:** a Department of Chemistry, J.J. Strossmayer University, Osijek, Franje Kuhača 20, HR-31000 Osijek, Croatia; b Laboratory of General and Inorganic Chemistry, Department of Chemistry, Faculty of Science, University of Zagreb, Horvatovac 102a, HR-10002 Zagreb, Croatia

## Abstract

The title mol­ecule, C_32_H_24_N_6_O_2_, has a crystallographic inversion centre in the middle of the benzodiimidazole core. It exists as the enol–imine tautomeric form and exhibits a strong intra­molecular O—H⋯N hydrogen bond. The dihedral angles between the planes of the 2-hy­droxy­phenyl and 4-amino­phenyl substituents and the plane of the benzodiimidazole unit [12.69 (8) and 84.71 (8)°, respectively] differ significantly due to steric reasons. In the crystal, mol­ecules are linked by C—H⋯π inter­actions, forming a two-dimensional network.

## Related literature

Benzodiimidazole and its derivatives are capable of adopting various coordination modes as well as forming multiple hydrogen bonds, see: Aakeröy *et al.* (2001 ▶); Holman *et al.* (2001 ▶). For the structures of benzodiimidazole derivatives with aromatic substituents, see: Boydston *et al.* (2006 ▶, 2007 ▶); Lin *et al.* (2004 ▶). For their pharmacological applications, see: Ansari & Lal (2009 ▶); Demirayak *et al.* (2011 ▶); Schulz & Skibo (2000 ▶). For applications of benzodiimidazole derivatives as ligands in coordination chemistry, see: Jiang *et al.* (2008 ▶). Some of their metal complexes have the property of metal-to-ligand charge-transfer excited states, see: Wang *et al.* (2011 ▶); Ohno *et al.* (1992 ▶).
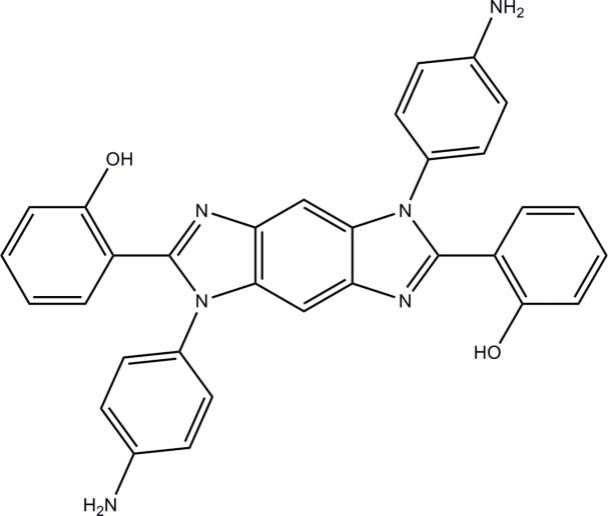


## Experimental

### 

#### Crystal data

C_32_H_24_N_6_O_2_
*M* *_r_* = 524.57
Monoclinic, 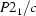

*a* = 6.5181 (3) Å
*b* = 13.3206 (7) Å
*c* = 14.3081 (7) Å
β = 91.680 (4)°
*V* = 1241.77 (11) Å^3^
*Z* = 2
Mo *K*α radiation
μ = 0.09 mm^−1^
*T* = 298 K
0.3 × 0.1 × 0.1 mm

#### Data collection

Oxford Diffraction Xcalibur CCD diffractometer
15503 measured reflections
2702 independent reflections
1605 reflections with *I* > 2σ(*I*)
*R*_int_ = 0.052

#### Refinement

*R*[*F* ^2^ > 2σ(*F* ^2^)] = 0.053
*wR*(*F* ^2^) = 0.123
*S* = 1.02
2702 reflections
190 parameters
H atoms treated by a mixture of independent and constrained refinement
Δρ_max_ = 0.17 e Å^−3^
Δρ_min_ = −0.16 e Å^−3^

### 

Data collection: *CrysAlis CCD* (Oxford Diffraction, 2003 ▶); cell refinement: *CrysAlis RED* (Oxford Diffraction, 2003 ▶); data reduction: *CrysAlis RED*; program(s) used to solve structure: *SHELXS97* (Sheldrick, 2008 ▶); program(s) used to refine structure: *SHELXL97* (Sheldrick, 2008 ▶); molecular graphics: *ORTEP-3* (Farrugia, 1997 ▶); software used to prepare material for publication: *WinGX* (Farrugia, 1999 ▶), *PARST97* (Nardelli, 1995 ▶) and *Mercury* (Macrae *et al.*, 2008 ▶).

## Supplementary Material

Crystal structure: contains datablock(s) I, global. DOI: 10.1107/S1600536811036737/kp2351sup1.cif

Structure factors: contains datablock(s) I. DOI: 10.1107/S1600536811036737/kp2351Isup2.hkl

Additional supplementary materials:  crystallographic information; 3D view; checkCIF report

## Figures and Tables

**Table 1:** Hydrogen-bond geometry (Å, °) *Cg* is the centroid of the C1–C6 ring.

*D*—H⋯*A*	*D*—H	H⋯*A*	*D*⋯*A*	*D*—H⋯*A*
O1—H1⋯N2	0.92	1.76	2.582 (2)	147
C14—H14⋯*Cg*^i^	0.93	2.60	3.514 (2)	167

Symmetry code: (i) 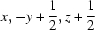.

**Fig. 1.**
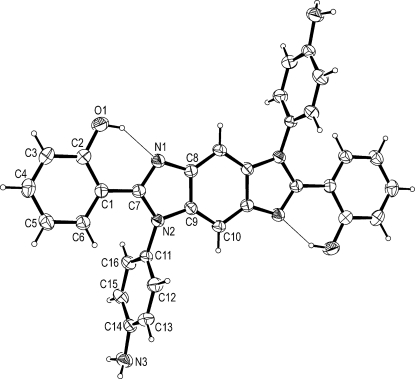
An ORTEPIII presentation of the molecule in a general orientation showing crystallographic numbering scheme. Anisotropic thermal ellipsoids are pictured with 30% of probability level.

**Fig. 2.**
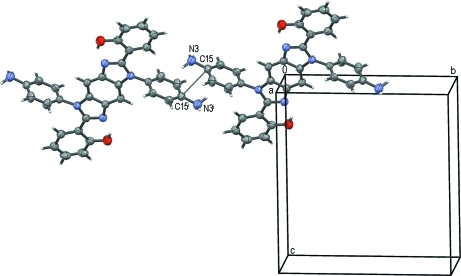
Two symmetry related molecules of (I), one in x, y, z and the another one in –x + 1, –y – 1, –z (i) position, showing spatial relationship between primary amino group N3 with C12-to-C17 aromatic ring.

**Fig. 3.**
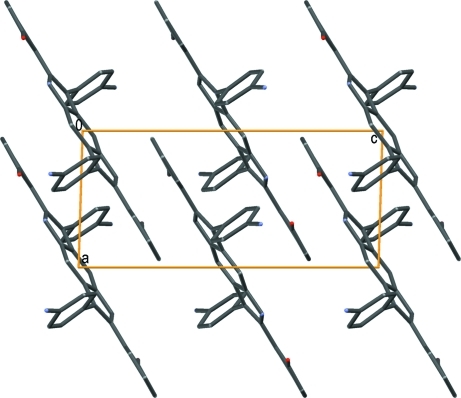
The projection down b-shows the orientation of symmetry related molecules in (000) and (001) planes, towards those in (001/2) plane.

## supplementary crystallographic information

### Comment

Benzodiimidazole and its derivatives are capable of adopting various coordination modes as well as forming multiple hydrogen bonds, which may provide a tool in crystal-engineering design for assembling building blocks into multi-dimensional structures (Aakeröy *et al.*, 2001; Holman *et al.*, 2001; Lin *et al.*, 2004; Boydston *et al.*, 2006; Boydston *et al.*, 2007; Jiang *et al.*, 2008). These compounds as potential complexing agents have been extensively investigated in recent years and were found to have a broad scope for spin crossover and biological activity. Benzodiimidazole and its derivatives are potential antitumor agents as inhibitors (Schulz & Skibo, 2000; Ansari & Lal, 2009; Demirayak, *et al.*, 2011) and some of their metal complexes have the property of metal-to-ligand charge-transfer excited states (Ohno *et al.*,1992; Wang *et al.*, 2011).

In this paper we report the synthesis and solid state structure of a novel heterocyclic system, the compound (I) containing benzodiimidazole core as a central moiety. The imidazole rings of later are substituted with 2–OH– and 4–aminobenzyl. Molecule is centrosymmetric with imposed inversion centre and placed in 2(*a*) special position of the space group *P*21/*c*. Molecular stereochemistry is defined by orientation of 2–OH– and 4–aminobenzyl substituent planes to the plane of benzodiimidazole (Fig. 1). Although 2-OH-benzyl is involved in forming of strong intramolecular O1–H···N2 hydrogen bond [O1-H···N2, 2.582 (2) Å] with benzodiimidazole moiety its plane deviates significantly from coplanarity with benzodiimidazole (interplanar angle 12.69°). Due to the spatial reasons the interplanar angle between the planes of 4–aminobenzyl and benzodiimidazole is 84.71°. A weak intermolecular hydrogen interactions C14–H14···π^i^ [3.514 Å (i): *x*, –*y–*1/2, *z* –1/2; π refers to the C1–C6 aromatic system centroid] linked molecules into a two-dimensional network. Surprisingly, primary N3 amino groups are not involved in significant intermolecular interactions. Nevertheless, weak attraction probably exists between primary amino group with C12-to-C17 aromatic ring at the distance slightly less than 3.5 Å (Fig. 2). Crystal packing is shown in Fig. 3.

### Experimental

The title compound has been prepared as a part of an investigation of the synthesis and characterisation of Schiff base ligands and their metal complexes. The compound (I) was derived from 1,5-dihydrobenzo[1,2 - *d*;4,5-*d'*]diimidazole in an attempt of template synthesis of copper(II) complexes with Schiff base ligand prepared from *p*-phenylenediamine and salycylaldehyde. It is known that metal Shiff base complexes have very low solubility and that it is hard to prepare appropriate single crystals for the X-ray structure analysis. For these reasons we examined the possibility of getting crystal suitable for X-ray analysis by slow synthesis reaction through liquid diffusion method. The expected compound was prepared in U-tube in such a way that one arm contained ethanolic metal-aldehydate solution (1 mmol of copper(II) chloride dihydrate and 2 mmol of salycilaldehyde) and the other one ethanolic diamine solution (2 mmol of *p*-phenylenediamine). Chloroform was in between two solutions. The resulting precipitate was orange plated crystals.

### Refinement

The position of hydrogen atoms bounded to N3 and O1 were located in the difference Fourier map and refined. Hydrogen atoms bounded to carbon were treated as riding atoms with C–H = 0.93 Å. Isotropic thermal parameters were set up as *U*_iso_(H) = 1.2 *U*_eq._

### Crystal data

**Table:**

C_32_H_24_N_6_O_2_	*F*(000) = 548
*M**_r_* = 524.57	*D*_x_ = 1.403 Mg m^−^^3^
Monoclinic, *P*2_1_/*c*	Mo *K*α radiation, λ = 0.71069 Å
Hall symbol: -P 2ybc	Cell parameters from 2702 reflections
*a* = 6.5181 (3) Å	θ = 4–27°
*b* = 13.3206 (7) Å	µ = 0.09 mm^−^^1^
*c* = 14.3081 (7) Å	*T* = 298 K
β = 91.680 (4)°	Prism, brown
*V* = 1241.77 (11) Å^3^	0.3 × 0.1 × 0.1 mm
*Z* = 2	

### Data collection

**Table:**

Oxford Diffraction Xcalibur CCD diffractometer	1605 reflections with *I* > 2σ(*I*)
Radiation source: fine-focus sealed tube	*R*_int_ = 0.052
graphite	θ_max_ = 27.0°, θ_min_ = 3.7°
ω scans	*h* = −8→8
15503 measured reflections	*k* = −16→17
2702 independent reflections	*l* = −18→18

### Refinement

**Table:**

Refinement on *F*^2^	Primary atom site location: structure-invariant direct methods
Least-squares matrix: full	Secondary atom site location: difference Fourier map
*R*[*F*^2^ > 2σ(*F*^2^)] = 0.053	Hydrogen site location: inferred from neighbouring sites
*wR*(*F*^2^) = 0.123	H atoms treated by a mixture of independent and constrained refinement
*S* = 1.02	*w* = 1/[σ^2^(*F*_o_^2^) + (0.0516*P*)^2^ + 0.1714*P*] where *P* = (*F*_o_^2^ + 2*F*_c_^2^)/3
2702 reflections	(Δ/σ)_max_ = 0.001
190 parameters	Δρ_max_ = 0.17 e Å^−^^3^
0 restraints	Δρ_min_ = −0.16 e Å^−^^3^

### Special details

**Table:**

Geometry. All e.s.d.'s (except the e.s.d. in the dihedral angle between two l.s. planes) are estimated using the full covariance matrix. The cell e.s.d.'s are taken into account individually in the estimation of e.s.d.'s in distances, angles and torsion angles; correlations between e.s.d.'s in cell parameters are only used when they are defined by crystal symmetry. An approximate (isotropic) treatment of cell e.s.d.'s is used for estimating e.s.d.'s involving l.s. planes.
Refinement. Refinement of *F*^2^ against ALL reflections. The weighted *R*-factor *wR* and goodness of fit *S* are based on *F*^2^, conventional *R*-factors *R* are based on *F*, with *F* set to zero for negative *F*^2^. The threshold expression of *F*^2^ > σ(*F*^2^) is used only for calculating *R*-factors(gt) *etc*. and is not relevant to the choice of reflections for refinement. *R*-factors based on *F*^2^ are statistically about twice as large as those based on *F*, and *R*- factors based on ALL data will be even larger.

### Fractional atomic coordinates and isotropic or equivalent isotropic displacement parameters (Å^2^)

**Table:**

	*x*	*y*	*z*	*U*_iso_*/*U*_eq_	
O1	−0.6794 (3)	0.55326 (12)	0.29326 (12)	0.0681 (5)	
H1	−0.549 (4)	0.5627 (18)	0.3187 (17)	0.082*	
N1	−0.2776 (2)	0.35831 (11)	0.44335 (11)	0.0445 (4)	
N2	−0.3419 (2)	0.51109 (12)	0.38381 (11)	0.0482 (4)	
N3	−0.3167 (4)	−0.02530 (16)	0.60297 (17)	0.0715 (6)	
H3A	−0.295 (4)	−0.077 (2)	0.5640 (19)	0.086*	
H3B	−0.424 (4)	−0.0300 (19)	0.6421 (19)	0.086*	
C1	−0.7197 (3)	0.45382 (16)	0.29194 (14)	0.0492 (5)	
C2	−0.5877 (3)	0.38185 (15)	0.33432 (13)	0.0446 (5)	
C3	−0.6341 (3)	0.28103 (16)	0.31968 (15)	0.0534 (6)	
H3	−0.5461	0.2325	0.3448	0.064*	
C4	−0.8066 (3)	0.25088 (17)	0.26907 (15)	0.0593 (6)	
H4	−0.8327	0.1830	0.2593	0.071*	
C5	−0.9399 (3)	0.32224 (19)	0.23309 (16)	0.0618 (6)	
H5	−1.0591	0.3024	0.2009	0.074*	
C6	−0.8976 (3)	0.42242 (18)	0.24457 (15)	0.0582 (6)	
H6	−0.9891	0.4700	0.2203	0.070*	
C7	−0.4052 (3)	0.41599 (15)	0.38676 (13)	0.0449 (5)	
C9	−0.1660 (3)	0.51705 (14)	0.44101 (13)	0.0444 (5)	
C10	−0.1229 (3)	0.42139 (14)	0.47848 (14)	0.0438 (5)	
C11	0.0420 (3)	0.40088 (14)	0.53848 (14)	0.0463 (5)	
H11	0.0676	0.3373	0.5630	0.056*	
C12	−0.3013 (3)	0.25779 (13)	0.47880 (13)	0.0410 (5)	
C13	−0.4253 (3)	0.24106 (15)	0.55365 (14)	0.0481 (5)	
H13	−0.5027	0.2932	0.5777	0.058*	
C14	−0.4337 (3)	0.14674 (16)	0.59246 (15)	0.0552 (6)	
H14	−0.5182	0.1358	0.6427	0.066*	
C15	−0.3202 (3)	0.06807 (15)	0.55872 (15)	0.0502 (5)	
C16	−0.2049 (3)	0.08554 (16)	0.48043 (17)	0.0604 (6)	
H16	−0.1349	0.0326	0.4535	0.072*	
C17	−0.1923 (3)	0.17967 (16)	0.44203 (16)	0.0560 (6)	
H17	−0.1099	0.1906	0.3910	0.067*	

### Atomic displacement parameters (Å^2^)

**Table:**

	*U*^11^	*U*^22^	*U*^33^	*U*^12^	*U*^13^	*U*^23^
O1	0.0738 (11)	0.0511 (10)	0.0777 (12)	0.0089 (8)	−0.0240 (9)	−0.0015 (8)
N1	0.0454 (9)	0.0404 (10)	0.0473 (10)	−0.0055 (7)	−0.0058 (8)	0.0064 (7)
N2	0.0500 (10)	0.0449 (11)	0.0492 (10)	−0.0047 (8)	−0.0061 (8)	0.0068 (8)
N3	0.0841 (15)	0.0528 (13)	0.0764 (16)	−0.0128 (11)	−0.0182 (12)	0.0168 (11)
C1	0.0499 (12)	0.0538 (14)	0.0438 (12)	0.0053 (10)	0.0007 (10)	−0.0017 (10)
C2	0.0428 (11)	0.0497 (13)	0.0414 (11)	−0.0026 (9)	0.0003 (9)	0.0031 (9)
C3	0.0566 (13)	0.0503 (13)	0.0527 (13)	−0.0070 (10)	−0.0088 (10)	0.0101 (10)
C4	0.0654 (14)	0.0585 (15)	0.0534 (14)	−0.0162 (12)	−0.0074 (11)	0.0032 (11)
C5	0.0514 (13)	0.0803 (18)	0.0532 (14)	−0.0080 (12)	−0.0085 (11)	−0.0006 (12)
C6	0.0496 (12)	0.0732 (17)	0.0513 (14)	0.0106 (11)	−0.0057 (10)	−0.0036 (11)
C7	0.0471 (11)	0.0445 (13)	0.0431 (12)	−0.0009 (9)	−0.0004 (9)	0.0047 (9)
C9	0.0456 (11)	0.0450 (12)	0.0424 (12)	−0.0014 (9)	−0.0015 (9)	0.0056 (9)
C10	0.0441 (11)	0.0420 (12)	0.0451 (12)	−0.0061 (9)	0.0002 (9)	0.0038 (9)
C11	0.0508 (11)	0.0381 (12)	0.0495 (12)	−0.0032 (9)	−0.0038 (9)	0.0102 (9)
C12	0.0434 (10)	0.0351 (11)	0.0441 (12)	−0.0027 (8)	−0.0042 (9)	0.0062 (9)
C13	0.0573 (12)	0.0428 (12)	0.0444 (12)	−0.0050 (9)	0.0048 (10)	−0.0067 (9)
C14	0.0681 (14)	0.0530 (14)	0.0447 (13)	−0.0141 (11)	0.0073 (11)	0.0008 (10)
C15	0.0524 (12)	0.0429 (13)	0.0544 (14)	−0.0091 (10)	−0.0129 (10)	0.0077 (10)
C16	0.0605 (13)	0.0451 (13)	0.0756 (17)	0.0090 (11)	0.0030 (12)	0.0007 (11)
C17	0.0539 (12)	0.0515 (14)	0.0634 (15)	0.0031 (10)	0.0168 (11)	0.0080 (11)

### Geometric parameters (Å, °)

**Table:**

O1—C1	1.350 (2)	C5—C6	1.372 (3)
O1—H1	0.92 (3)	C5—H5	0.9300
N1—C7	1.378 (2)	C6—H6	0.9300
N1—C10	1.395 (2)	C9—C11^i^	1.386 (3)
N1—C12	1.442 (2)	C9—C10	1.407 (3)
N2—C7	1.334 (2)	C10—C11	1.383 (3)
N2—C9	1.391 (2)	C11—C9^i^	1.386 (3)
N3—C15	1.395 (3)	C11—H11	0.9300
N3—H3A	0.90 (3)	C12—C17	1.373 (3)
N3—H3B	0.91 (2)	C12—C13	1.379 (3)
C1—C6	1.390 (3)	C13—C14	1.375 (3)
C1—C2	1.413 (3)	C13—H13	0.9300
C2—C3	1.391 (3)	C14—C15	1.378 (3)
C2—C7	1.460 (3)	C14—H14	0.9300
C3—C4	1.379 (3)	C15—C16	1.387 (3)
C3—H3	0.9300	C16—C17	1.372 (3)
C4—C5	1.377 (3)	C16—H16	0.9300
C4—H4	0.9300	C17—H17	0.9300
			
C1—O1—H1	108.4 (15)	N1—C7—C2	126.65 (17)
C7—N1—C10	107.06 (15)	C11^i^—C9—N2	129.41 (18)
C7—N1—C12	130.96 (15)	C11^i^—C9—C10	121.67 (17)
C10—N1—C12	121.10 (15)	N2—C9—C10	108.92 (17)
C7—N2—C9	106.63 (16)	C11—C10—N1	130.18 (17)
C15—N3—H3A	113.7 (17)	C11—C10—C9	123.91 (17)
C15—N3—H3B	109.8 (16)	N1—C10—C9	105.91 (16)
H3A—N3—H3B	118 (2)	C10—C11—C9^i^	114.41 (17)
O1—C1—C6	117.47 (19)	C10—C11—H11	122.8
O1—C1—C2	122.94 (19)	C9^i^—C11—H11	122.8
C6—C1—C2	119.6 (2)	C17—C12—C13	119.79 (18)
C3—C2—C1	117.61 (18)	C17—C12—N1	120.46 (17)
C3—C2—C7	123.25 (18)	C13—C12—N1	119.65 (17)
C1—C2—C7	119.06 (18)	C14—C13—C12	119.50 (19)
C4—C3—C2	122.0 (2)	C14—C13—H13	120.3
C4—C3—H3	119.0	C12—C13—H13	120.3
C2—C3—H3	119.0	C13—C14—C15	121.7 (2)
C5—C4—C3	119.4 (2)	C13—C14—H14	119.2
C5—C4—H4	120.3	C15—C14—H14	119.2
C3—C4—H4	120.3	C14—C15—C16	117.68 (19)
C6—C5—C4	120.3 (2)	C14—C15—N3	121.4 (2)
C6—C5—H5	119.9	C16—C15—N3	120.9 (2)
C4—C5—H5	119.9	C17—C16—C15	121.1 (2)
C5—C6—C1	120.9 (2)	C17—C16—H16	119.4
C5—C6—H6	119.6	C15—C16—H16	119.4
C1—C6—H6	119.6	C16—C17—C12	120.1 (2)
N2—C7—N1	111.48 (16)	C16—C17—H17	120.0
N2—C7—C2	121.87 (17)	C12—C17—H17	120.0

Symmetry codes: (i) −*x*, −*y*+1, −*z*+1.

### Hydrogen-bond geometry (Å, °)

**Table:**

Cg is the centroid of the C1–C6 ring.

**Table:**

*D*—H···*A*	*D*—H	H···*A*	*D*···*A*	*D*—H···*A*
O1—H1···N2	0.92	1.76	2.582 (2)	147
C14—H14···Cg^ii^	0.93	2.60	3.514 (2)	167

Symmetry codes: (ii) *x*, −*y*+1/2, *z*+1/2.
